# The Integration of AI-Driven Wearable Technology in Psychiatry: Advancing Early Detection and Personalized Management of Psychiatric Disorders

**DOI:** 10.1192/j.eurpsy.2025.391

**Published:** 2025-08-26

**Authors:** M. Peyioti, P. Argitis, A. Karampas, M. Demetriou, V. Anagnostopoulou, Z. Chaviaras

**Affiliations:** 1 University General Hospital of Alexandroupolis, Alexandroupolis; 2 Psychiatric Clinic, General Hospital of Corfu, Corfu; 3 Psychiatric Clinic, University General Hospital of Ioannina, Ioannina, Greece

## Abstract

**Introduction:**

Psychiatric disorders,such as anxiety,depression,bipolar disorder and schizophrenia,remain major global health challenges. Although prevalence has not recently increased, mental health care struggles with early diagnosis, real-time monitoring and personalized treatment. Traditional methods, relying on self-reports and clinical assessments,often miss the dynamic nature of these conditions.AI and wearable technology offer a new approach, enabling continuous data collection and real-time analysis to improve early detection and optimize patient care

**Objectives:**

This study aims to assess the role of AI-driven wearables in diagnosing,monitoring and managing psychiatric disorders by:

Evaluating AI’s effectiveness in predicting psychiatric episodes using wearable sensor data

Exploring clinical applications to improve patient outcomes

Identifying challenges and ethical considerations in the broader use of this technology in mental healthcare

**Methods:**

A systematic review of studies(2018-2023)on AI and wearable technology in psychiatry was conducted using PubMed, Scopus and Google Scholar.Studies were selected based on their focus on AI-driven wearables for predicting or managing psychiatric conditions.These devices typically captured physiological and behavioral data,such as heart rate variability,sleep patterns and movement.The accuracy of AI algorithms in predicting psychiatric episodes was compared to traditional methods,with statistical analysis used to assess outcomes

**Results:**

The review showed that AI-driven wearable devices significantly improved early detection and prediction of psychiatric episodes,with accuracy rates over 80% for depression, anxiety and bipolar disorder.Wearables,combined with AI algorithms, effectively monitored physiological data like heart rate and sleep patterns,providing real-time insights for personalized, timely interventions.For example,changes in sleep and activity levels,alongside heart rate variability,strongly predicted depressive episodes.In patients diagnosed with bipolar disorder,AI detected mood swings early by analyzing behavioral data from wearables,enabling stabilization.Wearables also helped monitor medication adherence and reduced relapse rates in patients diagnosed with schizophrenia by identifying early signs of psychotic episodes

**Conclusions:**

AI-driven wearable technology has the potential to transform psychiatric care by enabling continuous monitoring and personalized interventions.These tools enhance early detection and prediction of psychiatric episodes,offering a more dynamic approach than traditional methods.However,challenges such as data privacy, ethical concerns and the lack of regulatory frameworks must be addressed before widespread clinical use.Further research is needed to refine AI algorithms,validate the long-term effectiveness of wearables and ensure patient safety through regulations and privacy protections

**Disclosure of Interest:**

None Declared

